# Transient expression of the cancer/testis cancer antigen NY-ESO-1 in *Nicotiana benthamiana *using a PVX-based viral vector

**DOI:** 10.1186/1753-6561-8-S4-P76

**Published:** 2014-10-01

**Authors:** Cristiano Lacorte, Jéssica Carrijo, André Murad, Elíbio Rech

**Affiliations:** 1 Embrapa Recursos Genéticos e Biotecnologia, Estação Parque Biológica, 70770-910, Brasília, DF, Brazil; 2 Centro de Análises Proteômicas e Bioquímicas, Universidade Católica de Brasília, Brasília, DF, Brazil

## Background

Cancer/testis antigens is a group of proteins which expression is usually restricted to the human germ line but is also present at high levels in several types of cancer cells [1]. The antigen NY-ESO-1 is one of these antigens showing high immunogenicity and, therefore, holding great potential for a cancer vaccine [2]. Its heterologous expression is being tested in different expression systems, although the success has been limited [2,3]. In this report we used a transient expression system based on a PVX viral vector to evaluate the expression of NY-ESO-1 in leaves of *Nicotiana benthamina*.

## Methods

The NY-ESO-1 antigen coding sequence was synthetized based on a plant codon optimized sequence. The correspondent fragment was cloned in a pDonr207 derived vector containing a hexa-histidine tag (6His) and signal peptides for the endoplasmic reticulum (ER), apoplast or chloroplast. These vectors were recombined to a Gateway^®^-compatible PVX-based vector using the LR clonase, and transferred to *Agrobacerium tumefaciens *strain GV3101 containing the pSoup vector [4]. *Nicotiana benthamina *plants were inoculated with *Agrobacterium *suspensions through vacuum infiltration. After 4-5 days, leaves were collected and macerated in phosphate saline buffer (PBS) at a 1:2 (w:v) ratio and analyzed by Western blot.

To further characterize and quantify the expression levels of recombinant NY-ESO-1, leaf extracts were analyzed by nanoUPLC-MS^E ^[5]. To this end, leaves were grinded and proteins were extracted using 50mM Tris-HCl pH 8.0 in 1:20 (w:v) ratio. Proteins were precipitated with acetone, resuspended in water and quantified using the Qubit^® ^fluorimetric assay.

## Results and conclusions

The expression of cancer/testis NY-ESO-1 antigen was detected in *N. benthamiana *leaves using a PVX-based transient assay. Different constructs were tested containing signal peptides for ER, apoplast and chloroplast. No signal was detected when the NY-ESO-1 antigen was targeted to chloroplast or to the apoplast and detection was only possible when the protein was targeted to the ER, indicating that NY-ESO-1 accumulation in leaf tissue is influenced by the subcellular localization. NanoUPLC-MS^E ^analysis confirmed the presence of heterologous NY-ESO-1 in a level of approximately 0.1 % of the total soluble protein extract.

This assay allows protein to be detected after only 4-5 days after inoculation thereby eliminating the need of long and cumbersome tissue culture and selection schemes necessary for transgenic plants production. It also represents a handy and fast method to evaluate different gene constructs.

## References

[B1] Caballero OL Chen YT Cancer/testis (CT) antigens: potential targets for immunotherapy Cancer science 2009 100 11 2014 2021 19719775 10.1111/j.1349-7006.2009.01303.x PMC11158245

[B2] Simpson AJ Caballero OL Jungbluth A Chen YT Old LJ Cancer/testis antigens, gametogenesis and cancer Nature reviews 2005 5 8 615 625 10.1038/nrc1669 16034368

[B3] Lowe AJ Bardliving CL Huang CJ Teixeira LM Damasceno LM Anderson KA Ritter G Old LJ Batt CA Expression and purification of cGMP grade NY-ESO-1 for clinical trials Biotechnology progress 2011 27 2 435 441 21365782 10.1002/btpr.552

[B4] Lacorte C Ribeiro SG Lohuis D Goldbach R Prins M Potatovirus × and Tobacco mosaic virus-based vectors compatible with the Gateway cloning system Journal of virological methods 2010 164 1-2 7 13 19903495 10.1016/j.jviromet.2009.11.005

[B5] Murad AM Souza GH Garcia JS Rech EL Detection and expression analysis of recombinant proteins in plant-derived complex mixtures using nanoUPLC-MS(E) Journal of separation science 2011 34 2618 2630 21898799 10.1002/jssc.201100238

